# Multiple rhythms from one network: phase plane and stochastic analyses of rhythmic activity in turtle motor circuits

**DOI:** 10.1186/1471-2202-15-S1-P55

**Published:** 2014-07-21

**Authors:** Abigail Snyder, Jonathan Rubin

**Affiliations:** 1 Department of Mathematics, University of Pittsburgh, Pittsburgh, PA 15213, USA

## 

A central pattern generator (CPG) is a population of neurons producing rhythmic or repetitive behavior (i.e. scratching, walking) without requiring rhythmic input to the population. Turtles are observed to produce several rhythmic motor patterns in response to stimuli, in particular rostral scratch and pocket scratch (see Figure 1B inset) [1,2]. The rostral scratch and pocket scratch rhythms are created through the activity of three motoneurons: hip extensor, knee extensor, and hip flexor. A CPG (see Figure 1A) to produce both rhythms has been proposed, featuring a layer of populations of excitatory and inhibitory interneurons projecting to a layer of motoneurons [1]. We implement the CPG as a system of relaxation oscillators. The system successfully reproduces the desired rhythms by changing inputs to the layer of interneurons (see Figure 1B). We consider a dynamical systems approach to determine the mechanisms underlying rhythm generation. We analyze a proposed central pattern generator’s ability to produce differing motor patterns from a single pool of neurons under different tonic drives. A key issue is the knee extensor motoneuron’s response to different phasic synaptic inputs. We study the impact of these phasic inputs on motoneuron phase space and on properties of associated trajectories and show how these yield sufficient conditions for reproduction of observed rhythms. A contraction argument leads to existence of a stable solution. We also present preliminary results from stochastic analysis to examine the role of strong synaptic conductances.

**Figure 1:**
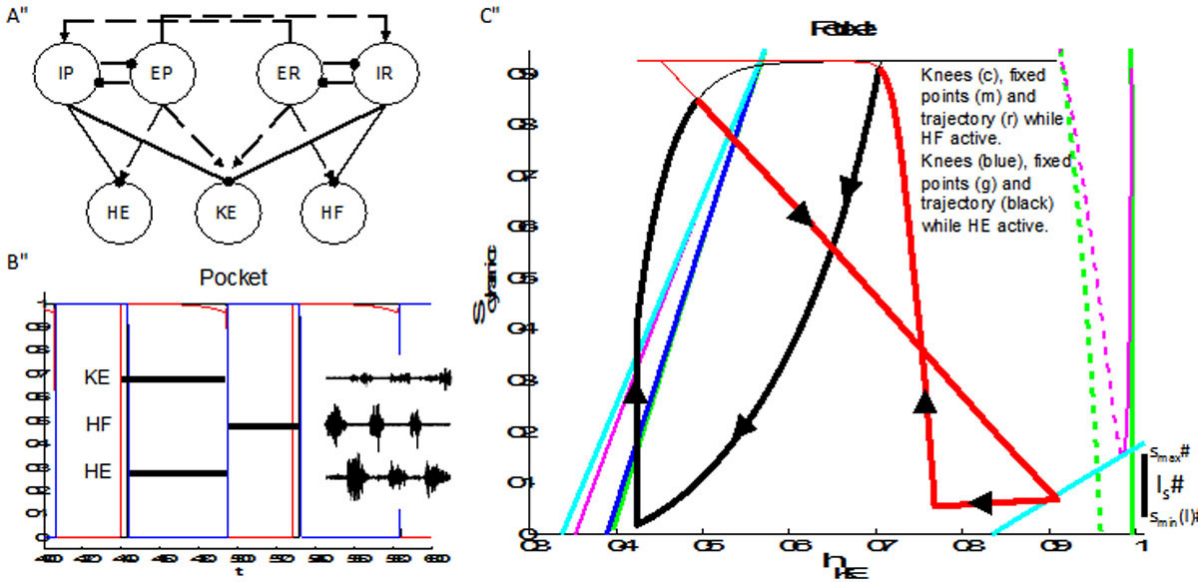
**A.** Implemented network architecture, adapted from [1] featuring a layer of populations of excitatory (EP, ER) and inhibitory (IP, IR) receiving external stimulation and driving a layer of populations of motoneurons (HE, KE, HF). Dashed arrows indicate excitatory synaptic connections, solid circles inhibitory; **B.** Example rhythm elicited under pocket stimulation. Inset is motor nerve recordings from [2]; **C.** Example reduced slow phase space trajectory for pocket rhythm.

## References

[B1] Berkowitz A Stein PSG Activity of descending propriospinal axons in the turtle hindlimb enlargement during two forms of fictive scratching: phase analyses J Neurosci 1994 15 8 5105 5119 8046471 10.1523/JNEUROSCI.14-08-05105.1994 PMC6577186

[B2] Berkowitz A Physiology and morphology of shared and specialized spinal interneurons for locomotion and scratching J Neurophysiol 2008 15 2887 9013 10.1152/jn.90235.2008 18385486

